# Assessment of Prostaglandin-Endoperoxide Synthase 2 and Versican gene expression profile from the cumulus cells: association with better in vitro fertilization outcomes

**DOI:** 10.1186/s13048-018-0456-2

**Published:** 2018-09-21

**Authors:** Azucena Ocampo, Jeimy Pedraza, Ginna Ortiz, Elizabeth Hernández-Pérez, Leonardo Porchia, Esther López-Bayghen

**Affiliations:** 1 Laboratorio de Investigación y Diagnóstico Molecular, Instituto de Infertilidad y Genética, Ingenes México, Av. IPN 2508, 07360 CDMX, México; 2 Departamento de Toxicología, Centro de Investigación y de Estudios Avanzados del Instituto Politécnico Nacional, Av. IPN 2508, 07360 CDMX, México; 3 Programa de Doctorado en Ciencias Biológicas y de la Salud, División de Ciencias Biológicas y de la Salud, Universidad Autónoma Metropolitana-Iztapalapa, Av. IPN 2508, 07360 CDMX, México

**Keywords:** Prostaglandin-endoperoxide synthase 2, Versican, IVF, Mexico, Cumulus cells

## Abstract

**Background:**

Current methods for determining superior embryo quality (morphological assessment) are unable to compensate for poor pregnancy outcomes. Due to the importance of the cumulus-oocyte complex and the value of cumulus cells (CCs) as markers of embryo health, we determined the association between the CCs gene expression of the Prostaglandin-Endoperoxide Synthase 2 (PTGS2) and Versican (VCAN) with pregnancy.

**Methods:**

One hundred forty-nine women, suffering from infertility and undergoing IVF, were included in this study (age: 29–46 years; BMI = 25.5 ± 5.0 kg/m^2^). Patients underwent a standard IVF protocol. CCs were isolated during oocyte retrieval, and their RNA was isolated using Trizol. The mRNA expression of PTGS2, VCAN, and L19 was measured by qPCR. The PVL index, (PTGS2 + VCAN)*L19_normalized_, was determined for each oocyte. Clinical pregnancy was confirmed by β-hCG and the presence of a fetal heartbeat. Associations were determined by ROC curves or logistic regression.

**Results:**

There was no correlation between the PVL index and morphological scores. Using only single embryo transfers (SETs), we determined that the PVL index was associated with pregnancy (β-hCG: AUC = 0.87, 95%CI: 0.74–1.00) with an optimal cutoff value of 58.2. Using the complete cohort (consisting of SETs, and patients with 2, 3, or 4 embryos transferred), the presence of at least one embryo with a PVL index score ≥ 58.2 was associated with a greater probability of achieving pregnancy (β-hCG: odds ratio = 17.15, 95%CI: 6.82–43.18, *p* < 0.001).

**Conclusion:**

Transferring at least one embryo with a PVL index score ≥ 58.2, generates a higher chance of achieving pregnancy.

## Background

Selection of embryos with a higher implantation potential is a significant challenge in Assisted Reproductive Technology. Currently, embryo selection is based mainly on morphological criteria such as growth rate, early cleavage on Day 2, the degree of fragmentation, and blastocyst formation, to name a few [1]; however, the predictive power of this approach remains limited. With the emergence of “Omics” technologies, new biomarkers can be diagnostic tools and utilized with in vitro fertilization (IVF) to improve oocyte and embryo selection [2].

A key factor in oocyte maturation is the cumulus-oocyte complex (COC), typically found in higher mammals. This complex results from the association between an oocyte and surrounding cumulus cells (CCs) through gap junctions [3]. Moreover, the development of competent oocytes highly depends on the bi-directional communication and interactions between the oocyte and CCs [4]. Several studies have investigated the association between CCs’ gene expression profiles with oocyte competence, embryo quality, and pregnancy outcomes using microarray, reverse transcriptase, and quantitative Real-Time PCR [5–10]. An indirect approach, using CCs RNA transcriptional data, was able to predict embryo quality and pregnancy outcome using gene expression signatures [11]. This raises the question of how can these technologies be used in determining embryo implantation potential. Measuring the expression levels of candidate biomarkers from the CCs can serve as a high-throughput, non-invasive approach to determine oocyte quality and successful pregnancy outcomes [8, 11]. However, there remains a need to determine the optimal usage and practical application of a particular set of genes to monitor and the evaluation of patient factors, such as age, etiology, insulin resistance, etc., that can affect these genes.

Previous studies have shown that Prostaglandin-Endoperoxide Synthase 2 (PTGS2) expression is associated with biological events, such as lesions, inflammation, and proliferation. A recent study demonstrated that up-regulation of PTGS2 in CCs of mice is associated with germinal vesicle to metaphase II (MII) stage transitions and oocyte competency [12]. In treated pigs, increased expression of PTGS2 resulted in improved oocyte competency [13]. In humans, PTGS2 expression in CCs is associated with the development of higher quality embryos [14]. Furthermore, PTGS2 expression levels are associated with good embryo morphology [15]. Many reports have convincingly established PTGS2 in oocyte maturation, nuclear maturation, and cumulus expansion, all predictors of clinical pregnancy [7, 16, 17]. However, even with the numerous studies showing PTGS2 importance, there are no studies to assess the predictability and practical use of PTGS2 levels of the CCs to estimate high-quality embryos that may achieve pregnancy.

Versican (VCAN) is a major component of the COC, located in the extracellular matrix, and its CCs gene expression has been reported as one of the most promising oocyte quality marker [6, 10, 18]. In mice, VCAN augmented a crucial step in embryonic development, cumulus expansion, and promoted the expression of PTGS [19]. In pigs, FSH stimulation increased the expression of VCAN in the CCs, promoting oocyte maturity [20]. In humans, oocyte quality has been positively associated with augmented VCAN expression [19, 21]. Lastly, the increased expression of VCAN at the oocyte stage resulted in a higher probability of pregnancy [10] and live births [18]. However, VCAN expression has yet to assess for its predictability and practical use as a marker of implantation and pregnancy.

Ranking of MII oocytes based on CC-expressed genes can serve as a promising new method for the selection of good quality MII oocytes derived from a pool of oocytes collected from hormone-stimulated IVF treatments in humans [22]. The CC-candidates genes, as potential oocyte quality markers for this study, were selected based on results from previous studies performed on human CCs [5, 14, 15, 18, 23] and were shown to be involved in the process of cumulus expansion, prediction of embryo development, and pregnancy. Therefore, the purpose of this study was to assess the VCAN and PTGS2 gene expression in CCs from individual COC as markers of oocyte quality and predictors of clinical pregnancy, in a manner that could be useful to the IVF laboratory as an extra tool to choose the best combination in number and quality at transfer time.

## Results

### Participants and study characteristics

One hundred and ninety-eight women that suffer from infertility undergoing IVF in Mexico City, Mexico were selected for this study. Some subjects were lost due to not returning for follow-ups appointments, failure to produce viable oocytes/embryos, failure to collect sufficient RNA from the CCs for analysis, or chose not to be included. CCs RNA was isolated from the individual oocytes for each IVF cycle; however, 2 IVF cycles failed to produce a signal for PTGS2, VCAN, and L19, whereas 9 IVF cycles failed to produce a signal for PTGS2, VCAN, or both, while L19 gene was amplified. Therefore, the study consisted of 31 patients who had a single embryo transferred (SETs), 41 patients with two embryos transferred, 68 patients who had three embryos transferred, and nine patients who had four embryos transferred. Clinical and IVF characteristics are presented in Table 1.

**Table 1:** Characteristics of study participants

Category	Value
Sample size (n)	149
Age (years)	37.3 ± 4.9
Body-mass index (kg/m^2^)	25.2 ± 4.2
≥7 cells/Day 3 (n)	7.5 ± 1.8
Embryo fragmentation (%)	8.7 ± 4.3
Fertilization rate (%)	65.2 ± 18.0
Embryos transferred (n)	2.4 ± 0.9
1 embryo	31
2 embryos	41
3 embryos	68
4 embryos	9
Pregnancy rate (%)	59.89 ± 13.74
Etiology	
Endometrioma (n)	5
Points of endometriosis (n)	10
Low response (n)	3

### There is no correlation between the PVL index and the embryo morphological assessment scores

**Fig. 1:**
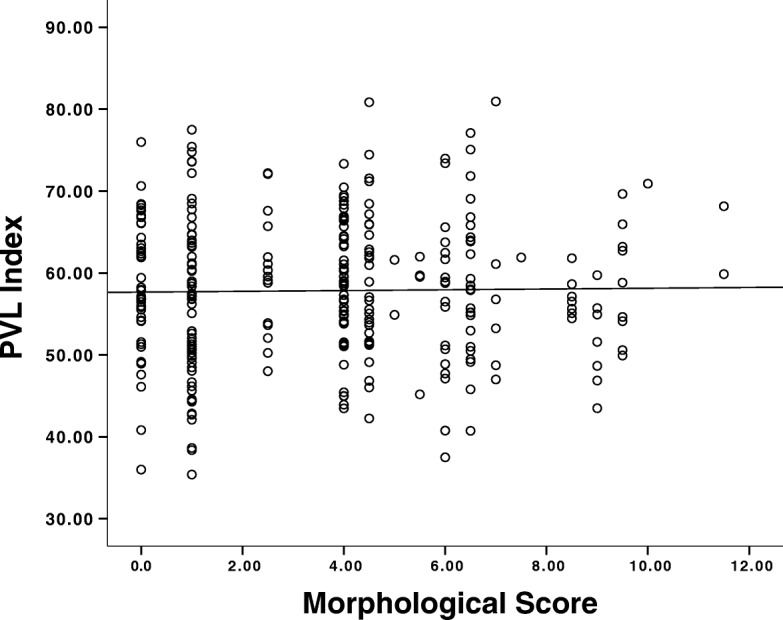
Correlation between embryo morphological score and the PVL index. For 42 patients, CCs were isolated from an oocyte during a standardized IVF protocol. CCs RNA was isolated using Trizol. Gene expression profile of PTGS2, VCAN, and L19 was determined by qPCR and the PVL index was calculated. Afterwards, oocytes were fertilized and morphological parameters evaluated by a specialized training Embryologist for 384 embryos. The level of association was determined by calculating Spearman’s correlation coefficient (ρ)

A subset of 42 patients agreed to have their complete embryo cohort that consisted of high and low-quality embryos, as determined by morphological assessment, assessed. Of the 384 embryos analyzed, the morphological assessment of these embryos ranged between 0 and 12, whereas the PVL index scores ranged between 35.4 and 80.9. There was no association between the PVL index and the embryo morphological assessment scores (ρ = − 0.013, *p* = 0.831, Fig. 1).

## PTGS2 and VCAN gene expression levels in CCs are associated with clinical pregnancy

**Fig. 2:**
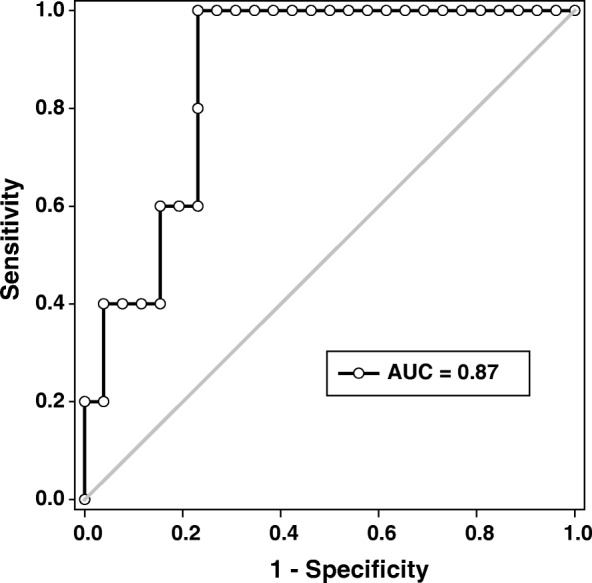
Receiver operating characteristic curve for the PVL index to clinical pregnancy. 31 single embryos transfers were implanted and pregnancy was confirmed by β-hCG level > 10 mUI/ml on Day 14. The PVL index was determined for each oocyte

Based on their highest morphological score, only high-quality embryos were selected for implantation. Between one and four embryos were transferred per patient (Table 1); however, the effectiveness of the PVL index was assessed using the 31 SETs. ROC analysis determined that the PVL index was highly predictive for implantation (AUC = 0.87, 95% CI: 0.74–1.00, *p* = 0.010, Fig. 2). Using the highest Youden’s index, we determined that a PVL score ≥ 58.2 was associated with clinical pregnancy (Youden index = 0.769, sensitivity = 100%, and specificity = 76.9%) and was highly accurate (test accuracy = 80.65%, positive predictive value = 45.5%, and negative predictive value =100%).

The effectiveness of the PVL index was also examined in patients with multiple embryos implanted using the 58.2 cutoff value. All IVF cycles (*n* = 149), which consisted of SETs, two embryos transferred, three embryos transferred, and four embryos transferred, were evaluated. To correct for embryo cohorts lacking a completely positive or negative PVL group, we used a modified equation proposed by Ekart et al. and calculated the probability of pregnancy for each IVF cycle. Using logistic regression, a strong association was determined between the probability of pregnancy based on the PVL index and implantation (β-hCG: odds ratio = 11.59, 95%CI: 4.27–31.48, *p* < 0.001), as well as ultra-sound confirmed presence of fetal sac with a heartbeat (odds ratio = 8.40, 95%CI: 3.26–21.63, *p* < 0.001, Table 2).

**Table 2:** The association between the *probability* and pregnancy determined by β–hCG or USG

Category	Size	OR ^a^	95% CI	*p*-value
Pregnancy by β–hCG				
Probability raw score^b^				
Overall	149	11.59	4.27–31.84	< 0.001 *
Patient’s age				
< 38 years of age	63	11.46	2.16–60.65	0.004 *
≥ 38 years of age	86	12.41	3.48–44.21	< 0.001 *
Pre-implantation Genetic Testing (PGT)				
No PGT confirmed euploid embryo	104	6.81	2.03–22.86	0.002 *
PGT confirmed euploid embryo	45	51.23	6.46–406.25	< 0.001 *
Patient’s health				
No major etiology	131	17.43	5.48–55.44	< 0.001 *
Presence of a major etiology	18	4.48	0.19–104.06	0.350
At least 1 PVL positive embryo ^c^				
Overall	149	17.15	6.82–43.18	< 0.001 *
Patient’s age				
< 38 years of age	63	13.60	3.71–49.91	< 0.001 *
≥ 38 years of age	86	21.93	5.73–83.94	< 0.001 *
Pre-implantation Genetic Testing (PGT)				
No PGT confirmed euploid embryo	104	9.67	3.54–26.43	< 0.001 *
PGT confirmed euploid embryo	45	N/D		
Patient’s health				
No major etiology	131	29.17	10.43–81.56	< 0.001 *
Presence of a major etiology	18	1.20	0.09–15.26	0.888
Pregnancy by Ultrasound confirmation				
Probability raw score ^b^				
Overall	149	8.40	3.26–21.63	< 0.001 *
Patient’s age				
< 38 years of age	63	3.84	0.92–16.12	0.066
≥ 38 years of age	86	14.79	4.08–53.61	< 0.001 *
Pre-implantation Genetic Testing (PGT)				
No PGT confirmed euploid embryo	104	6.31	1.94–20.50	0.002 *
PGT confirmed euploid embryo	45	21.62	3.53–132.42	0.001 *
Patient’s health				
No major etiology	131	10.75	3.77–30.65	< 0.001 *
Presence of a major etiology	18	4.48	0.19–104.06	0.350
At least 1 PVL positive embryo ^c^				
Overall	149	16.81	6.43–43.92	< 0.001 *
Patient’s age				
< 38 years of age	63	9.48	2.66–33.78	0.001 *
≥ 38 years of age	86	31.63	6.68–149.79	< 0.001 *
Pre-implantation Genetic Testing (PGT)				
No PGT confirmed euploid embryo	104	10.27	3.64–28.96	< 0.001 *
PGT confirmed euploid embryo	45	N/D		
Patient’s health				
No major etiology	131	26.91	9.29–77.94	< 0.001 *
Presence of a major etiology	18	1.20	0.09–15.26	0.888

^a^Crude odds ratio (OR) and 95% confidence intervals (95% CI) were determined using logistic regression. N/D = was not able to be determined. *indicates a significant result, *p* < 0.05 (two-tailed). ^b^Probability = 1-(x_neg_/x_tot_)^n^, where x_neg_ = number of embryos with a PVL index score < 58.2, x_tot_ = total number of transferred embryos, and *n* = number of embryos with a PVL index score ≥ 58.2. ^c^A positive cohort has the probability ≠0.00

Interestingly, the implantation of at least one embryo with a PVL index score ≥ 58.2, independent of the total number of embryos implanted, was associated with a greater chance of achieving clinical pregnancy, as determined by β-hCG (Odds Ratio = 17.15, 95%CI: 6.82–43.18, Table 2) and ultra-sound confirmed presence of fetal sac with a heartbeat (Odds Ratio = 16.81, 95%CI: 6.43–43.92, *p* < 0.001, Table 2). Using all 149 IVF cycles, the PVL index was highly accurate (test accuracy = 78.52%, positive predictive value = 76.0%, and negative predictive value =84.4%).

When the group was stratified by age and considering the transfer of at least 1 PVL positive embryo, there was an increased association for women ≥38 years of age for β-hCG (0.6-fold change) and for ultrasound confirmation (2.3-fold change, Table 2). For many of the embryos, PGT was used to confirm the embryos that were transferred were euploid. When stratified by the PGT-confirmed absence of aneuploidy embryos, there was an increased association for β-hCG (6.5-fold change) and for ultrasound confirmation (2.4-fold change) using the probability’s raw score. When considering the transfer of at least 1 PVL positive embryo, the fold change could not be determined due to the lack of false negatives for the PGT confirmed euploid embryos. Lastly, 18 subjects were identified with severe etiologies (endometriosis or low response during stimulation) other than infertility. When these patients were stratified, the association between the PVL index and pregnancy was only present in the subjects absent from these etiologies.

## Discussion

Gene expression analysis of the CCs can be a valuable tool that allows attaining an estimation of oocyte quality and embryo capabilities, especially in respect to embryo implantation. Different groups have analyzed the CCs transcriptional profile, resulting in the assembly of a group of candidate genes of which only a few have been suggested as genes that could predict oocyte quality and pregnancy success [5–8, 10, 18, 23–25]. For this study, two genes expressed in the CCs were analyzed: PTGS2 and VCAN. Even though both genes have been highly reported in the literature for their association to oocyte quality, to date, neither gene has been included in a single panel profile used to evaluate clinical pregnancy potential for embryos. Here, we show that a high PVL index, which is evaluating the expression of these two genes, resulted in increased clinical pregnancy. Many groups have assessed the gene profile of the CCs, all indicating that both genes, PTGS2 and VCAN, play an important role in oocyte maturation and are relevant indicators of competent oocytes [6, 14, 15, 18]. Gebhardt et al. revealed the presence of a positive correlation with embryonic development and live births. Regardless that Gebhardt et al. proposed PTGS2 and VCAN as candidate genes to measure oocyte quality and embryonic development [18], there is no evidence in the literature to support their practical use in oocyte and embryo selection. In this study, these genes were therefore proposed as the first group of genes that can be used in conjunction with morphological data produced by the IVF laboratory for the selection of embryos to be transferred. A transcriptional profile was generated for the CCs using qPCR data, which allowed us to generate an expression indicator, the PVL index, for the evaluation of oocyte quality and embryo capabilities to implant. Here, only high-quality embryos were selected and transferred. Afterward, their respective PVL index scores were assessed. We determined that the PVL index scores were independent of the morphological assessment. This does demonstrate that the cellular processes vary significantly between similarly scored embryos and posits that alternative tests are required when selecting embryos.

It is imperative to emphasize that until today, no reports establish a cutoff value, based on gene expression, where competent and not competent oocytes/embryos were considered. This study proposes the application of an index that relates the expression of VCAN and PTGS2, as a new tool for the evaluation of pregnancy prediction. Using SETs, which were only high-quality embryos, the PVL index, and measurements of clinical pregnancy presented with a good correlation. This led to establishing a cutoff value for the PVL index of 58.2 (Fig. 2). Afterward, IVF cycles with multiple embryos transferred were assessed, and it was determined that the cutoff value for the PVL index was highly predictive. Unfortunately, there were minimal IVF cycles with completely positive (≥58.2) or completely negative (< 58.2) PVL index score embryos. Therefore, it is difficult to determine if the embryos with the higher PVL index scores are the ones producing the pregnancy when mixed cohorts and several embryos are transferred. Even though, we demonstrated that the implantation of at least one embryo with a PVL score ≥ 58.2 was associated with clinical pregnancy.

Ekart et al. was one of the first groups to propose a new classification and selection system for oocytes, based on the genetic expression shown in the CCs, specifically using molecules involved in the COC interaction that are activated during the second to last phase of folliculogenesis. In addition of developing a mathematical tool that can be applied for oocyte selection, their system allows an evaluation of the expression, followed by expression level classification of four genes from the CCs: *hyaluronan synthase 2* (HAS2), *follicle-stimulating hormone receptor* (FSHR), VCAN, and *progesterone receptor*. Combination of the HAS2 and FSHR genes resulted in a predictive value of 80% when applying for the selection of three embryos. However, using this system for a single embryo selection, the predictive value decreased significantly to 48%. Ekart et al. did not include PTGS2 in their gene panel to predict oocyte quality and embryonic development [22]. Even though the PVL index was used to score each embryonic cohort, showing a strong correlation between this index and clinical pregnancy, an additional mathematical analysis was performed to support our findings. The mathematical formula created by Ekart was applied to each embryonic cohort. In theory, this would determine the probability of each embryo to produce a clinical pregnancy, only if this embryo was from an oocyte with a CCs quality index ≥58.2. Undeniably, the probability of pregnancy of a transferred embryo displayed a high correlation with the PVL index and therefore aids us in predicting pregnancy in patients.

Older women have a decreased probability of achieving pregnancy and lower IVF success rates; therefore, exploiting alternative methods to improve IVF outcome remains a key factor. When the cohort was stratified by age, the PVL index was more associated with older women in achieving clinical pregnancy. This posits that using the PVL index could improve the probability of successful implantation for older women.

The implantation of aneuploid embryos is associated with lower IVF success rates and the level of aneuploidy in embryo-cohorts increases with age. In Mexico, older women are suggested to complement IVF with PGT, to assess for aneuploidy; however, the benefits and pitfalls of using of PTGS2 remains under debate. Here, only 30% of the patients opted to have PGT; therefore, it is possible that some of the embryos were genetically compromised, as shown by the decreased diagnostic odds ratio when we examined embryos without confirmed euploidy. Unfortunately, with the embryos that were determined to be euploid, we were unable to determine the diagnostic odds ratio, when at least 1 PVL positive embryo was implanted. This was due to the absence of any false negative results. In other words, the presences of a PVL positive embryo was not associated with failed implantation. This posits that using both the PVL index and PGT would improve IVF outcomes.

Our study has a few limitations. First, we focused on a random set of females with some level of primary and secondary female infertility factor—male factor was not considered. We can only speculate that male factor infertility will not affect the results demonstrated here, as the examined genes are from the CCs and only associated with oocyte health and competence. Second, some of the patients had endometriosis of varying degree, which was probably affecting the implantation results. Endometriosis and its location could affect and explain why some patients did not present with the clinical pregnancy even with good embryos. However, this is outside the scope of the current research and is currently being considered for future studies. Lastly, we cannot be entirely confident if the high PVL scored embryos were the embryos that achieved clinical pregnancy, but SETs results support our confidence in this possibility. This is the preliminary study, and the selection of embryos based on the PVL index is the focus of current and future studies.

## Conclusions

The development of new tools, which allow us to obtain an approximation of the state of the oocyte and the embryo, as well as determine the clinical pregnancy potential, is of great importance for IVF treatments. Here, a valuable evaluation system was generated to measure two key genes from the CCs—PTGS2 and VCAN—to relate the ovular state and clinical pregnancy. The PVL index can indicate good quality oocytes that for after fertilization will have the highest probability of achieving clinical pregnancy. This research will allow embryologists and other IVF personnel involved in the selection process to have an alternative test to determine the best embryo to transfer over the current method of embryo morphological assessment.

## Methods

### Study patients and ethical approval

Women that suffer from infertility, undergoing IVF in Mexico City, Mexico, were asked to participate in this retrospective study (from October 2011 to May 2017). The protocol was approved by the Ethics Committee of the Ingenes Institute (number I/13/2013). Written informed consent was obtained from all patients, conducted in accordance with the Declaration of Helsinki. Patients were clinically evaluated according to a standardized protocol including personal and family clinical history. The patients’ height (m) and weight (kg) were measured, and the BMI was calculated as weight divided by the height squared (kg/m^2^).

### IVF, CC isolation, and pregnancy evaluation

All patients were subjected to controlled ovarian stimulation for ten days with Gonadotropin-releasing hormone agonists and antagonists. Ovarian response was assessed measuring serum estradiol levels, and follicular development was evaluated by ultrasound examination. Oocyte retrieval was conducted 20 h after human chorionic gonadotropin (hCG) administration (10,000 IU Choragon or 6500 IU Ovidrel) with ultrasound guidance. Follicular puncture for oocyte collection was performed under general anesthesia at the end of hormonal stimulation (10–14 days). Transvaginal ultrasound was used to locate mature follicles, and ovulation was induced with hCG. 3–5 ml of follicular fluid containing the oocytes were extracted using a specialized suction system. Follicles aspirated from the patients ranged between 8 and 30. Samples were analyzed using a stereoscopic microscope in order to locate the oocytes, which were kept at 37.5 °C in an atmosphere of 8.3% CO_2_ until fertilization. Number and quality of retrieved oocytes were assessed using morphological parameters [granulosa expansion, oocyte maturity (MI, MII, and VG), quality of the cytoplasm, zona pellucida, and polar body]. Oocytes were located, numbered, and separated into drops of HTF-HEPES (Human tubal fluid/(4-(2-hydroxyethyl)-1-piperazineethanesulfonic acid) supplemented with 10% HSA. CCs were removed using a 1-mm needle and isolated using mechanical dispersion. The isolated CCs were preserved in 20 μl of Global Total for Fertilization media (LifeGlobal) and placed into Eppendorf tubes containing 150 μl of Trizol (Ambion, Life Technologies, Carlsbad CA, USA) and stored at − 70 °C until processed. To note, the patient’s cumula cells were not pooled; the oocyte and its CCs were analyzed as a corresponding pair.

An Embryologist monitored and recorded information about fertilization, embryo development, embryo morphology, transfer, and pregnancy for each oocyte. Morphological parameters evaluated were weighed into a matrix to rate each oocyte-embryo, with the sum of values obtained on a scale of 0 (low quality) to 12 (high quality). Selection and embryo transfer were done on Day 3 or Day 5 of development according to the embryo morphological assessment, using the criteria established by Istanbul consensus Workshop on Embryo Assessment [26]. The highest quality embryos (morphology) were transferred, and pregnancy was confirmed by β-hCG values > 10 mUI/ml (Day 14) and the presence of a fetal heartbeat, confirmed by ultrasound at 6–8 weeks. The number of embryos transferred (1, 2, 3, or 4) was determined by the number of high-quality embryos achieving full development, patient results from previous attempts, and the opinion of the clinician.

### RNA extraction

CCs RNA extraction was carried out using the Trizol® reagent, according to the manufacturer’s recommendations. Briefly, CCs samples were processed with 70 μl of chloroform for 5 min at room temperature, followed by centrifugation at 12,500 g for 15 min at 4 °C. The supernatant was transferred to a new tube containing 150 μl isopropanol. Samples were incubated for 10 min at room temperature and centrifuged at 15,000 g for 15 min at 4 °C. The pellet was washed with 100 μl of 75% ethanol and then centrifuged at 12,500 g for 5 min. Pellet was air-dry for 10 min. RNA was re-suspended in 0.1% of DEPC water and quantified by spectrophotometry (Epoch/Biotek, Winooski, VT, USA).

### Quantitative reverse transcription- polymerase chain reaction (RT-qPCR)

Primers for PTGS2, VCAN, and Ribosomal Protein L-19 (L19) were designed using the Primer 3 plus v2.0 software. All primer sequences are shown in Table 3. All qPCR reactions were performed using the StepOne Plus apparatus (Applied Biosystems) with the One Step Kappa Syberfast system (KAPA Biosystems, Woburn, MA, USA). PTGS2, VCAN, and L19 genes were quantified in duplicate. Reaction mix was prepared as follows: 5 μl 2X KAPA SYBR® FAST qRT-PCR Master Mix, 0.2 μl ROX, 0.2 μl dUTP (10 mM), 0.2 μl forward and reverse primers (20 pmol), 0.2 μl KAPA RT, 100 ng of RNA sample, and DEPC water for a total volume of 10 μl. qPCR conditions were one cycle of reverse-transcription at 42 °C for 5 min, one cycle of reverse-transcriptase inactivation at 95 °C for 5 min, 40 cycles of amplification at 95 °C for 15 s, 56 °C for 30 s, then 72 °C for 30 s. SYBR Green was used during amplification to construct melting curves that were analyzed to verify if the peaks corresponded with theoretical melting temperatures for each amplicon. All the PCR products were resolved through capillary electrophoresis using the BioAnalyzer Labchip GX (Caliper). The products showed a single band corresponding to the predicted base pair length and a band purity of 95% or higher. Moreover, the bands were cloned and analyzed via sequencing to verify their identity. Sequence identification of the PCR products was confirmed by direct cloning with the CloneJet system (Fermentas, ThermoFisher, Waltham, MA, USA) and sequencing using the BigDye system. Briefly, the amplicon fragments were purified using the GeneJet Gel Extraction kit and ligated into a pJET1.2/blunt vector following the manufacturer’s protocol. Ligations were transformed into TOP10 competent bacteria and grown in LB medium (Ampicillin, Pisa SA Laboratorios, Mexico; 100 mg/ml) for 16 h at 37 °C. Plasmid DNA was extracted from the bacteria using the mini-prep technique. Amplicon’s identity was verified by sequencing using BigDye Terminator v3.1 reagent and the RV primer 3 (3’-CTAGCAAAATAGGCTGTCCC-5′) (Applied Biosystems, Foster City, CA, USA). Samples were sequenced with the ABI PRISM 3700 analyzer (Applied Biosystems) and sequences corroborated using Blast software.

**Table 3:** Primer sequences

Gene	Forward Primer	Reverse Primer
PTGS2	5’-CTGAAGCCCTATGAATCATTT-3′	5’-CATTACCCATAAGTCCTTTCAA-3′
VCAN	5’-TCAGCAAAGGACAATTCAATA-3′	5’-TTTAAAATGTTTTGGGAGCA-3′
L19	5’-TCAGGCTACAGAAGAGGCTTGC-3′	5’-ATCAGCCCATCCTTGATCAGC-3′

### PTGS/VCAN/L19 (PVL) index and probability

The PVL index was calculated from the data obtained after processing the isolated CCs of each individual oocyte. L19 was determined to be the optimal housekeeping gene for our system as the variations associated with L19 in a cohort were no larger than 1 C_T_ in more than 90% of cases. For each patient, the L19 expression was used for normalizing purposes (to the lowest C_T_ for the patient’s oocyte cohort). The index is the sum of the expression levels of PTGS2 and VCAN, normalized by L19.

1 $$ \mathrm{PVL}\ \mathrm{Index}=\left(\left[\mathrm{PTGS}2\right]+\left[\mathrm{VCAN}\right]\right)/{\left[\mathrm{L}19\right]}_{\mathrm{normalized}} $$

The probability of pregnancy for each transfer cycle using the PVL index was obtained by the modified formula for random selection described by Ekart et al. [22]:

2 $$ \mathrm{P}=1-{\left({\mathrm{x}}^{\mathrm{n}\mathrm{eg}}/{\mathrm{x}}^{\mathrm{tot}}\right)}^{\mathrm{n}} $$

where P = probability, x^neg^ = number of embryos with a PVL index score < 58.2, x^tot^ = total number of transferred embryos, and n = number of embryos with a PVL index score ≥ 58.2.

### Embryo biopsy (day 3 and day 5)

Embryos were assessed for the number of cells, symmetry, and fragmentation. For high-morphological quality embryos, the chromosomal composition was determined using Array Comparative Genomic Hybridization (aCGH). The S-biopsy method was utilized to isolate a blastomere from Day 3 embryos [27]. Briefly, a Hamilton Thorne ZILOS-tk laser (1460 nm, 300 mW) was used to create a funnel in the zona pellucida adjacent to a blastomere. Next, the blastomere was extracted by aspirating the whole embryo with a 140-μm stripper capillary micropipette, leading to the ejection of the blastomere. The blastomere was then placed into a 0.2-μL PCR tube. For Day 5 embryos (expanded blastocyst stage containing 50 to 150 cells), a Hamilton Thorne ZILOS-tk laser was used to create a funnel in the zona pellucida on the opposite side to the inner cell mass. Blastocysts were incubated for a further 2–3 h to allow blastocele expansion and herniation of the trophectoderm cells from the zona pellucida. Afterward, the embryo was placed into 20-μL of Ca^2+^/Mg^2+^-free bicarbonate buffered G-PGD medium (cat #10074, Vitrolife). Applying gentle suction with the biopsy pipette (MBB-FP-SM-35, Origio, Malov, Denmark), the trophectoderm cells were encouraged to herniate from the zona pellucida. Trophectoderm cells were dissected from each of the blastocysts using four laser pulses of 3-min duration. 10–15 cells were retrieved, washed, and placed into a 0.2-μL PCR tube.

### Whole genome amplification and pre-implantation genetic testing (PGT)

The material obtained from each biopsy was amplified using the SurePlex amplification system (Illumina, San Diego, CA, USA) according to the manufacturer’s instructions. PGT was carried out by aCGH using the 24 Sure V3 microarray (Illumina, San Diego, CA, USA) using the protocol described by Fragouli [28, 29]. The amplified DNA was fluorescently labeled (Fluorescence Labelling System, Illumina). The samples were co-precipitated, denatured, and analyzed by array hybridization (for 16 h). A laser scanner (InnoScan 710, Innopsys, Carbonne, France) was used to excite the fluorophores and read the hybridization images. Hybridization images were stored in TIFF format and analyzed by the BlueFuse Multi-Analysis software (Illumina), using the criteria and algorithms recommended by the manufacturer. With this approach, it was possible to determine the chromosome constitution of each embryo.

### Statistical analysis

The association between the PVL index and embryo morphological assessment scores was determined by calculating Spearman’s rho (ρ). Receiver operating characteristic (ROC) analysis was performed to determine the specificity and sensitivity of the PVL index, by calculating the area under the ROC curve (AUC). The cutoff value was determined calculating the highest Youden Index score: sensitivity + specificity–1. Logistic regression was used to determine the association (Odds Ratio and 95% confidence intervals) between the PVL index and clinical pregnancy. *P*-values < 0.05 (two-tailed) were considered significant. All analyses were carried out using either the Statistical Package for the Social Sciences program, version 22 (SPSS, Chicago, IL) or Sigma Plot software (v. 12.0, San Jose, CA, USA).

## Abbreviations

aCGH: array Comparative Genomic Hybridization
AUC: Area under the ROC curve
CCs: Cumulus cells
COC: Cumulus-oocyte complex
FSHR: *Follicle-stimulating hormone receptor*
HAS2: *Hyaluronan synthase 2*
IVF: In vitro fertilization
PGT: Pre-implantation genetic testing
PTGS2: *Prostaglandin-Endoperoxide Synthase 2*
PVL index: (PTGS2 + VCAN)*L19_normalized_
qPCR: Quantitative Reverse Transcription- Polymerase Chain Reaction
ROC: Receiver operating characteristic
SETs: Single embryo transfers
VCAN: *Versican*
β-hCG: Beta human Chorionic Gonadotropin
